# De Novo Design of Miniprotein Inhibitors of Bacterial Adhesins

**DOI:** 10.21203/rs.3.rs-7951484/v1

**Published:** 2025-11-04

**Authors:** Adam Chazin-Gray, Tuscan Thompson, Edward Lopatto, Pearl Magala, Patrick Erickson, Andrew Hunt, Anna Manchenko, Pavel Aprikian, Veronika Tchesnokova, Irina Basova, Denise Sanick, Kevin Tamadonfar, Morgan Timm, Jerome Pinkner, Karen Dodson, Alex Kang, Emily Joyce, Asim Bera, Aaron Schmitz, Ali Ellebedy, Kelli Hvorecny, Gianluca Interlandi, Mark Cartwright, Andyna Vernet, Sarai Bardales, Desmond White, Rachel Klevit, Evgeni Sokurenko, Scott Hultgren, David Baker

**Affiliations:** University of Washington; University of Washington; Washington University in St. Louis; University of Washington; University of Washington; University of Washington; University of Washington; University of Washington; University of Washington; University of Washington; Washington University in St. Louis; Washington University School of Medicine; Washington University School of Medicine; Washington University School of Medicine; Washington University School of Medicine; University of Washington; University of Washington; University of Washington; Washington University in St. Louis; Washington University School of Medicine; University of Washington; University of Washington; Harvard University; Harvard University; Harvard University; Harvard University; University of Washington School of Medicine; University of Washington; Department of Molecular Microbiology and Center for Women's Infectious Disease Research, Washington University School of Medicine; University of Washington

## Abstract

The rise of multidrug-resistant bacterial infections necessitates the discovery of novel antimicrobial strategies. Here, we show that protein design provides a generalizable means of generating new antimicrobials by neutralizing the function of bacterial adhesins, which are virulence factors critical in host-pathogen interactions. We de novo designed high-affinity miniprotein binders to FimH and Abp chaperone usher pili adhesins from uropathogenic Escherichia coli and Acinetobacter baumannii, respectively, which are implicated in mediating both uncomplicated and catheter-associated urinary tract infections (UTI) responsible for significant morbidity worldwide. The designed antagonists have high specificity and stability, disrupt bacterial recognition of host receptors, block biofilm formation, and are effective in treating and preventing murine models of uncomplicated and catheter-associated UTIs in vivo.

Multidrug-resistant (MDR) bacterial pathogens constitute a significant and growing threat to public health (1). To counter the alarming rise of these increasingly untreatable and deadly bacterial infections, accelerating the development of novel antimicrobial therapies is essential. Recent advances in protein design have opened the door to designing small (< 120 amino acids) *de novo* protein binders (minibinders) that precisely target virtually any epitope (2,3), enabling therapeutics against viruses (4), toxins (5), and other disease targets (6). However, a major challenge in applying these tools to combat bacteria is the accessibility of minbinders to intracellular and membrane-embedded target antigens due to their shielding by surface molecules such as lipopolysaccharides. Previous attempts have identified high-affinity protein binders to their integral outer membrane protein target antigens *in vitro*, only to find that the binders cannot access their targets in a cellular context (7,8).

We reasoned that bacterial adhesins would constitute ideal targets for designed minibinder therapeutics targeting bacteria because they lie well beyond bacterial surface molecules and play a key role in the virulence of MDR bacteria by mediating their attachment to and invasion of host epithelial cells as well as biofilm formation (Fig. 1a). Among the diverse family of bacterial adhesins, chaperone-usher pathway (CUP) pili adhesins lie at the tips of extracellular fibers, and recognize receptors in a lectin-substrate interaction that mediates host tropisms. CUP pili are ubiquitous among gram-negative bacteria and are critical for the establishment and persistence of numerous bacterial infections, including both uncomplicated urinary tract infections (UTIs) and catheter-associated UTIs (CAUTIs; Fig. 1a). UTIs are one of the most common bacterial infections among women and their treatment accounts for approximately 15% of antibiotic usage in the United States (9,10). Over 50% of UTIs, especially those acquired in hospitals, are caused by MDR bacteria, limiting the effectiveness of a dwindling number of traditional antibiotic treatments (11–13). Previous anti-adhesin therapeutic strategies have relied on small molecule ligand mimetic inhibitors (14) or monoclonal antibodies (mAbs) (15,16). However, these strategies require identification of the adhesin substrate, which is often unknown, or labor-intensive screening for inhibitory mAbs. Designed minibinders could overcome this therapeutic bottleneck by enabling the rapid development of antibiotic-sparing therapies, relying solely on the adhesin structure and identification of its putative binding pocket. To investigate this, we sought to design minibinders against three CUP adhesins: FimH from *Escherichia coli*, which accounts for 70–90% of UTIs, and Abp1D and Abp2D from *Acinetobacter baumannii*, which is prevalent in hospital-acquired CAUTIs and is often multidrug resistant.

## Results

### *De novo* design of minibinders targeting FimH

Uropathogenic *E. coli* (UPEC) expresses type 1 pili, a prototypical CUP pilus, which are tipped with the mannose binding adhesin FimH (17). UPEC FimH is essential for bacterial attachment to the bladder epithelium via mannosylated glycoproteins (18). After attachment, UPEC forms intracellular bacterial communities that are recalcitrant to antibiotics and, if left untreated, can lead to recurrent UTIs and kidney infection (18). FimH consists of an N-terminal mannose-binding lectin domain and a C-terminal pilin domain that attaches to the rest of the pilus (18,19). The lectin domain is an allosteric protein that samples a conformational equilibrium between two well-characterized conformational states: a low-affinity state (LAS, also known as the “Tense” state) and a high-affinity state (HAS, also known as the “Relaxed” state) (20). In the LAS, the body of the FimH lectin domain interacts with the pilin domain, fixing it in place, and allosterically disrupts the mannose binding pocket, resulting in a low affinity for mannose (18,19). In the HAS, the lectin domain has little to no interaction with the pilin domain, allowing it to rotate freely relative to the pilin domain, and the mannose binding pocket loops are stably positioned to bind to mannose (18).

**Figure 1:**
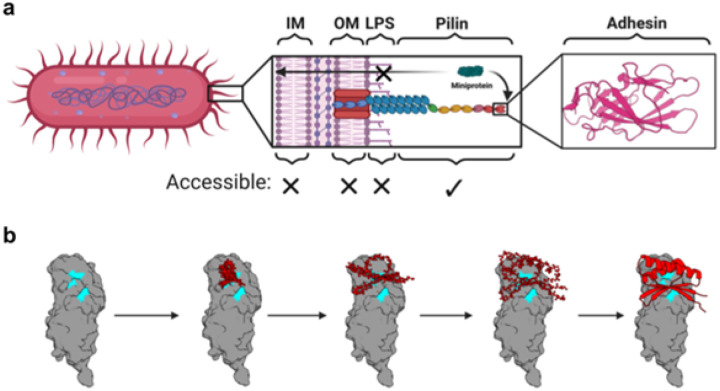
*De novo* design of adhesin inhibitors. **(A)** Schematic illustrating the structures on the gram-negative bacterial cell surface that are potentially accessible to protein-based inhibitors. Virulence factors that make up the bacterial pili, especially tip adhesins, are attractive targets due to their essential role in pathogenesis and the fact that they lie well off the membrane and beyond the protective lipopolysaccharide shell. Created with BioRender.com. **(B)** Example denoising trajectory for an Abp2D binder. “Hotspots” corresponding to the adhesin substrate binding pocket residues are highlighted in cyan.

We used RFdiffusion (3) to generate novel minibinder backbones against both states of FimH. We targeted designs to the binding pocket of published crystal structures (FimH in LAS: 3JWN; FimH in HAS: 1UWF) by specifying “hotspot” residues in the binding pocket as inputs to the model (Fig. 1b). We subsequently assigned sequences to these backbones using ProteinMPNN (21) and scored designs with AlphaFold2 (AF2) Initial Guess (22). The best sequences ranked by AF2 folding and interaction confidence metrics (predicted local distance difference test (pLDDT) and predicted aligned error (PAE) scores) were then iteratively refined by partially diffusing (23) the AF2 predicted structures with RFdiffusion, reassigning sequences with ProteinMPNN, and filtering with AF2. We obtained an oligonucleotide array encoding the top 10,000 designs by AF2 and Rosetta interface metrics.

### Experimental characterization of FimH minibinders

FimH minibinders were screened via cDNA display, rather than a cell-based screening approach like yeast surface display, because FimH binds to mannose on the cell surface. We panned against the wild-type FimH lectin domain, which adopts the HAS, and a mutant lectin domain stabilized in the LAS (24). The top 96 designs enriched more than four-fold over the naive library and consisted of 78 binders identified through the HAS sorts and 39 binders identified through the LAS sorts. Twenty-one designs were enriched against both states. Eighty-one of the enriched sequences were designed against the LAS.

**Figure 2:**
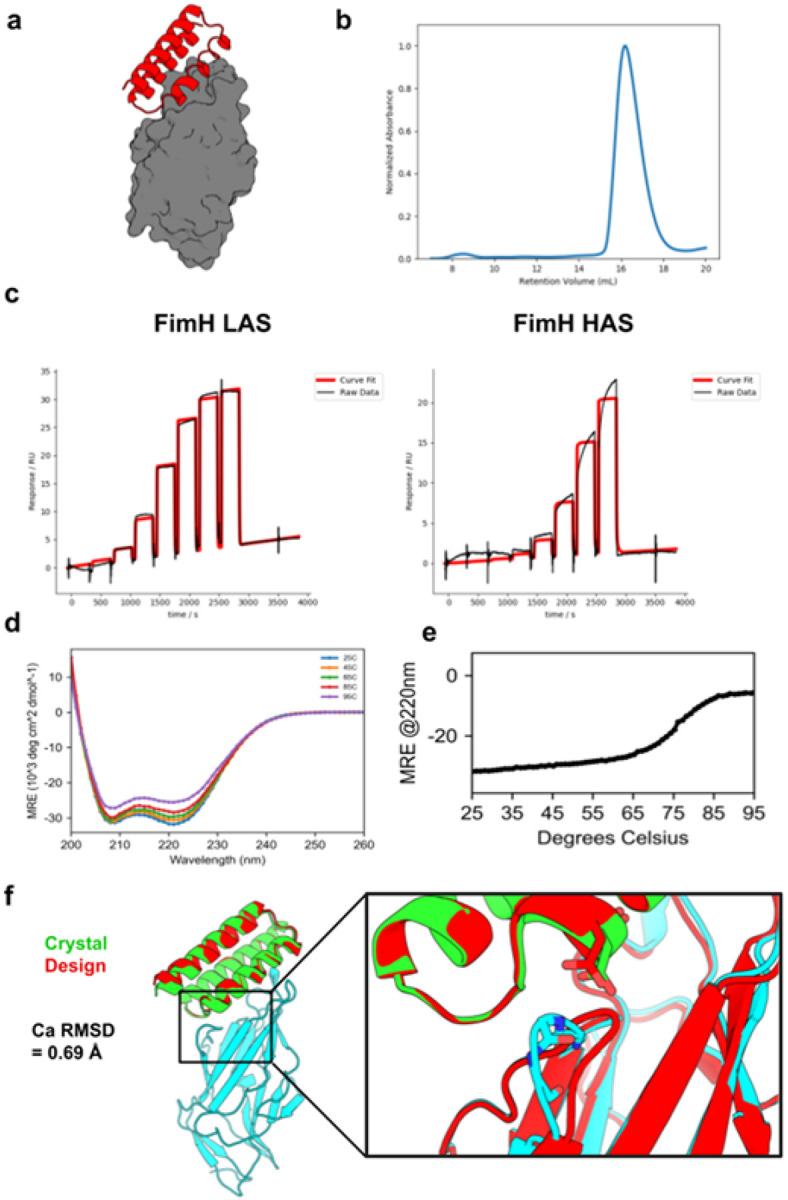
*In vitro* characterization of FimH minibinder F7. **(A)** Design model for FimH minibinder F7 (red) in complex with FimH LAS (gray). **(B)** SEC trace of F7: sample was injected onto a Superdex 75 10/300 GL column. **(C)** The binding affinity of F7 was determined with SPR. The SPR data indicate that F7 binds more tightly to the LAS (left; K_d_=119 nM) than HAS (right; K_d_=713 nM). **(D)** Raw CD data for F7. Full wavelength scans from 250 to 200 nm were performed at the indicated temperatures. **(E)** A melting curve for F7 indicates that it is thermostable up to 75°C. CD signal at 220 nm (helicity) was measured every 1°C. **(F)** Crystal structure of F7 (green) with FimH L34K (cyan) overlaid on the design model (red); inset: difference in FimH clamp loop between design and crystal structure showing an unexpected displacement of the clamp loop. The experimental structure exhibits a Ca RMSD of 0.69 Å to the design model.

We expressed these 96 FimH minibinders in *E. coli* and found that 79 exhibited monodisperse size exclusion chromatography (SEC) traces and expressed sufficiently for downstream characterization (see Materials and Methods for details). In an initial surface plasmon resonance (SPR) binding screen, nine of these designs exhibited sub-micromolar binding against at least one of the FimH states (table S3). All nine designs were originally designed against the LAS. To directly assess the ability of FimH minibinders to inhibit and disrupt bacterial adhesion, we performed a series of red blood cell (RBC) aggregation assays using *E. coli* strain KB23 expressing type 1 fimbriae. *E. coli* aggregates RBCs by binding to mannosylated glycoproteins on their surface via FimH. Of the 96 minibinders we tested, eight significantly inhibited RBC aggregation (Fig. S1), seven of which also had submicromolar affinities in the SPR screen. Minibinder F7 had the lowest minimum inhibitory concentration (69 nM) in the RBC aggregation inhibition assay and was therefore selected for further characterization (Fig. S2). F7 was designed against the LAS, expressed well in *E. coli*, and purified as a monodisperse species (Fig. 2a, 2b). Though it was designed against the LAS, F7 was identified by panning against the HAS. Further characterization by SPR confirmed that F7 binds to both conformations, but with a several-fold higher affinity to the LAS (K_d_=119 nM) than HAS (K_d_=713 nM) (Fig. 2c). F7 exhibited the expected circular dichroism (CD) spectra and proved to be highly thermostable, with a melting point above 75°C (Fig. 2d, 2e).

Co-crystallization of F7 with a mutant FimH lectin domain stabilized in the LAS (L34K) (25) confirmed the designed binding interface with a C*a* RMSD of 0.69 Å to the design model (PDB Code: 9Q1V; Fig. 2f). The experimentally determined structure of FimH bound to F7 was much closer to the canonical LAS conformation than the HAS conformation (C*a* RMSD of 0.45 Å vs 2.26 Å). As expected, the minibinder occluded the binding pocket of FimH. Unexpectedly, it also wedged between one of the mannose-binding loops and the clamp loop, further displacing it from the binding pocket compared to the design model. The clamp-loop displacement induced by F7 closely resembles that of the recently determined structure of FimH bound to anti-FimH antibody mAb926, which was raised against the LAS and known to function through a parasteric mechanism (fig. S3) (26).

Given that F7 preferentially binds the LAS-stabilized mutant FimH and that the crystal structure of FimH in complex with the minibinder resembles the LAS, we suspected that F7 binding may induce a shift from the HAS to the LAS in wild-type FimH lectin domain. We compared F7’s interaction with the L34K variant of the lectin domain (LAS) and wild-type lectin domain (HAS) by (^15^N, ^1^H)-HSQC NMR spectroscopy. The substantial structural differences between the LAS and HAS conformations of the isolated lectin domain are reflected in their distinct NMR spectra, which display very little peak overlap (fig. S4A, S4B). Addition of F7 to the LAS variant caused chemical shift perturbations localized at the binding interface, consistent with a binding event and/or local change in conformation, but not a global conformational change (fig. S5A). In contrast, the spectrum of the WT HAS domain showed widespread chemical shift changes, becoming similar to that of the LAS variant (fig. S5A). Notably, spectra of the F7-bound HAS and LAS variants now show extensive peak overlap, indicating a shared conformation (fig. S4C). Together, these data indicate that F7 binding to the isolated WT lectin domain induces a conformational change from the HAS to the LAS, shifting the conformational equilibrium in the opposite direction as binding the native ligand and existing mannoside therapeutics (20).

**Figure 3:**
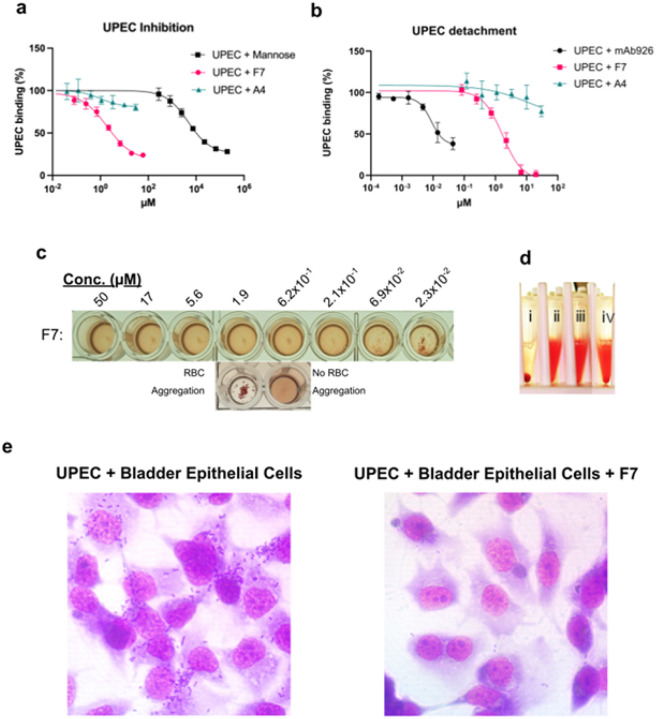
*In cellulo* characterization of FimH minibinder F7. **(A)**Inhibition ELISA results for minibinder F7 (pink; IC50=1.9 μM; 95% CI: 1.4–2.9 μM), noninhibitory minibinder A4 (teal), and mannose (black). Each experiment includes three replicates. Error bars show standard deviation. **(B)** Detachment ELISA results for F7 (pink; IC50=1.7 μM; 95% CI: 1.3–2.4 μM), A4 (teal), and mAb926 (black). Each experiment includes three replicates. Error bars show standard deviation. **(C)** Red blood cell aggregation inhibition assay with a serial dilution of F7 (MIC=69 nM). **(D)** Red blood cell disaggregation assay showing red blood cells incubated for one hour with bacteria (i), without bacteria (ii), with bacteria and 2% mannose (iii), incubated for 1 hour with bacteria and then for 15 minutes with minibinder F7 (iv). **(E)** Effect of F7 on bacterial adhesion to bladder epithelial cells. The addition of F7 decreases the number of bacteria present.

We quantitatively assessed the ability of minibinder F7 to block bacterial adhesion to surfaces coated with bovine RNAseB—a model glycoprotein rich in Man_5_ high-mannose type oligosaccharides that avidly binds FimH even in the LAS. We preincubated *E. coli* KB23 with either F7, alpha-methyl-mannoside, or a minibinder that showed no inhibition in RBC assays (A4) and then measured its binding to RNaseB. F7 displayed an IC50 of 1.9 μM (95% CI: 1.4–2.9 μM), a more than two-thousand-fold improvement over mannose (Fig. 3a). We then performed a detachment ELISA by first adhering bacteria to RNAseB-coated plates and subsequently introducing either F7, noninhibitory minibinder A4, or mAb926, which is known to effectively dissolve preformed biofilms (27). F7 but not A4 successfully detached UPEC with an IC50 of 1.7 μM (95% CI: 1.3–2.4 μM), but fell short of mAb926 by two orders of magnitude (Fig. 3b). We also sought to characterize F7’s inhibitory ability in a series of more biologically relevant RBC assays. F7 successfully inhibited RBC aggregation by *E. coli* with a minimum inhibitory concentration of 69 nM (Fig. 3c), exceeding by more than a thousand-fold that of alpha-methyl-mannoside (fig. S6). We tested the ability of F7 to reverse bacterial aggregation by adding it to pre-aggregated mixtures of RBC and *E. coli*. F7 successfully redispersed the RBCs within 15 minutes of introduction at 1 μg/ml (Fig. 3d). We confirmed the physiological relevance of F7's inhibitory ability by comparing the adherence of bacteria to T24 urinary bladder tissue culture cells in the presence and absence of minibinder. F7-treated cells had considerably fewer bacteria present than either untreated cells (Fig. 3e) or cells treated with A4 (fig. S7).

Given the sequence diversity of FimH across disease-causing strains, we tested whether F7 is inhibitory not only to the FimH variant it was designed against but also to different FimH variants expressed by the most common highly-virulent and/or multi-drug resistant clonal groups of UPEC. F7 successfully inhibited RBC aggregation by strains from the clonal groups ST73, ST95, ST69, and ST131-H30 (fig. S8, table S4). F7 also inhibited aggregation by *E. coli* strain cas665 expressing *Klebsiella pneumoniae* FimH, which has approximately 85% sequence identity to *E. coli* FimH, suggesting that F7 may be cross-reactive with FimH variants from other bacterial pathogens.

### *De novo* design of minibinders targeting Abp adhesins

CAUTIs caused by MDR bacteria pose a significant economic toll on the healthcare system in the United States (28). CAUTIs represent approximately 40% of hospital-acquired infections and have an increased risk of morbidity and mortality compared to uncomplicated UTIs (28,29). CAUTIs result in a serious deterioration of quality of life through pain, discomfort, disruption of daily activities, and increased healthcare costs, exacerbated by the rapid spread of drug resistance in uropathogens (28–32).

One particularly concerning CAUTI pathogen is *A. baumannii*, which is classified by the CDC as a pathogen of urgent concern due to its high rates of multidrug resistance and increases in relative prevalence in CAUTIs compared to uncomplicated UTIs (33). The *A. baumannii* strain “ACICU” possesses two highly similar CUP pili, Abp1 and Abp2, that play critical roles in mediating CAUTI. The Abp1 and Abp2 pili are tipped with the Abp1D and Abp2D adhesins that allow these bacteria to bind to host-deposited fibrinogen on implanted catheters, which is a critical step for *A. baumannii* to establish a CAUTI (34). While the receptor binding domains of these adhesins share only 70% sequence identity, they share a higher degree of structural homology as seen in previously solved crystal structures (Abp1D: 8DF0; Abp2D: 8DEZ).(34) Previous structure-function studies identified the putative fibrinogen-binding pockets of these adhesins; however, the exact structural binding interactions between fibrinogen and the adhesins remain unknown (34). Similar to the dynamic conformational ensembles displayed by FimH, the binding affinity of the *A. baumannii* Abp1D and Abp2D receptor binding domains (RBD) are controlled via dynamics of the flexible anterior binding loop, illustrated by comparing the loop’s conformation in the Abp2D crystal structure to an AlphaFold2 model for this protein (fig. S9). In addition, while vaccination with Abp2D protected mice from *A. baumannii* CAUTI, these efforts to immunize mice with Abp2D failed to identify sufficiently inhibitory mAbs to effectively disrupt binding of ACICU to fibrinogen (IC50s > 100 μM) in a bacterial ELISA assay (fig. S10) (35). Thus, we were motivated to apply protein design tools to develop more effective Abp inhibitors (36).

We used RFdiffusion (3) with “hotspot” conditioning to generate novel minibinder backbones specifically targeting the putative fibrinogen-binding sites of both the Abp1D and Abp2D adhesins (Fig. 1b). Taking into account the anterior loop conformational differences between the high-affinity and low-affinity states, we designed backbones against the crystal structures of Abp1D and Abp2D receptor binding domains, which display the anterior binding loop in the open (low-affinity) conformation, exposing the putative binding pocket. We assigned sequences to these backbones using ProteinMPNN, predicted their structures with AF2 Initial Guess, and then iteratively optimized high-quality designs as described above. The top ~5000 scoring designs against Abp1D (~1300) and Abp2D (~3700), with a maximum of 15 sequences per backbone, were obtained as a DNA oligo chip.

### Experimental characterization of Abp minibinders

Abp adhesins do not bind mannose and are therefore amenable to screening minibinders using yeast surface display. Designed Abp binder sequences were amplified from the DNA oligo chip and cloned into a yeast display expression vector using yeast cloning. The resulting yeast display library was enriched for binding via two rounds of fluorescence-activated cell sorting (FACS) with 1 μM biotinylated Abp1D or Abp2D lectin domains using PE-conjugated streptavidin as a detection reagent. A third round of FACS sorting involved staining the enriched populations with 10-fold titrations of these adhesins from 1000 nM to 1 nM, revealing strong binding signal for the Abp2D-enriched population down to 1 nM, and binding signal down to 10 nM for the Abp1D-enriched population (fig. S11). Next-generation sequencing of sorted populations identified 31 highly enriched binder sequences across both populations, including some sequences that were enriched in both Abp1D and Abp2D sorts, suggesting that we identified cross-reactive binders. Interestingly, all 31 of these enriched binders were designed using the Abp2D crystal structure, which might be explained by the presence of a citrate molecule in the Abp2D crystal structure that opens its putative binding pocket and exposes more hydrophobic residues than in the Abp1D structure.

**Figure 4:**
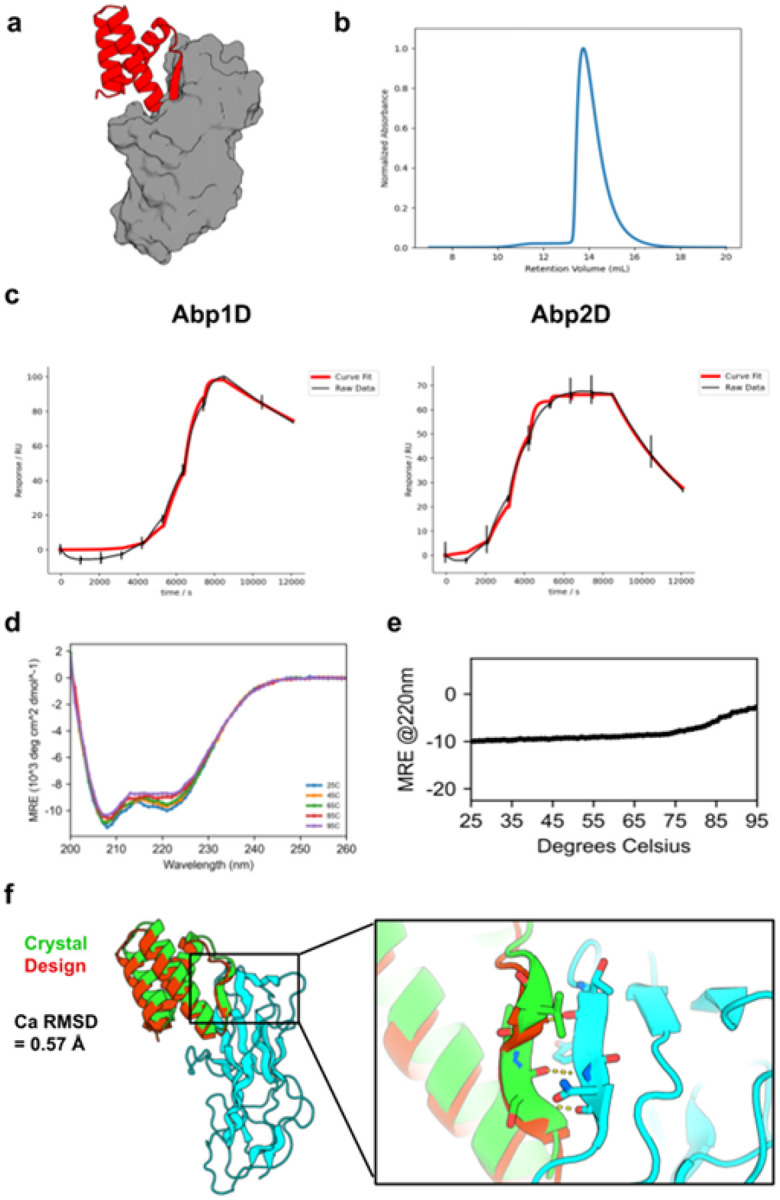
*In vitro* characterization of Abp minibinder A7. **(A)** Design model for Abp minibinder A7 (red) in complex with Abp2D (gray). **(B)** SEC trace of A7: sample was injected onto a Superdex 75 10/300 GL column. **(C)** The binding affinity of A7 was determined with SPR. The SPR data indicate that A7 binds with high affinity to Abp1D (left; K_d_=50.4 nM) and Abp2D (right; K_d_=3.5 nM). **(D)** Raw CD data for A7. Full wavelength scans from 260 to 200 nm were performed at the indicated temperatures. **(E)** A melting curve for A7 indicates that it is thermostable up to 80°C. CD signal at 220 nm (helicity) was measured every 1°. **(F)** Crystal structure of A7 (green) with Abp2D (cyan) overlaid on the design model (red); inset: beta-strand pairing interaction between A7 and an edge strand of Abp2D. The experimental structure exhibits a Ca RMSD of 0.88 Å to the design model.

Minibinder sequences that were enriched during yeast surface display with Abp adhesins were expressed in and purified from *E. coli*. Of the 31 enriched binders, twenty-four exhibited primarily monodisperse SEC traces and had sufficient yields for downstream characterization. An initial SPR binding screen of these 24 binders against both Abp1D and Abp2D identified 16 binders that exhibited sub-micromolar affinity to Abp2D, four of which also exhibited sub-micromolar affinity to Abp1D (table S5). A follow-up SPR experiment on the four cross-reactive binders revealed that Abp minibinder A7 (design model in Fig. 4a) exhibited high affinity to both Abp1D (K_d_=50.4 nM) and Abp2D (K_d_=3.5 nM; Fig. 4c). A7 expressed well (~50 mg/L of culture), behaved as a monodisperse species as assessed by SEC (Fig. 4b), exhibited the expected CD spectra (Fig. 4d), and was thermostable with a melting temperature greater than 85°C (Fig. 4e). Next, we confirmed the specificity of the designed adhesin binders using SPR. Minibinder A7 exhibited undetectable levels of binding to FimH LAS or HAS. Similarly, FimH minibinder F7 exhibited insignificant binding to Abp1D and Abp2D (fig. S12).

Determining the structure of a co-crystal of A7 with Abp2D confirmed the designed interface, and exhibited a C*a* RMSD of 0.88 Å to the design model (PDB Code: 9Q1H; Fig. 4f). This structure confirms the designed binding mode that utilizes a helix-turn-strand motif to simultaneously stabilize the core of the minibinder, insert a loop deep into the putative fibrinogen binding pocket of Abp2D, and strand pair with an edge strand of this adhesin. As designed, A7 stabilizes the anterior binding loop of Abp2D in an open conformation, thus neutralizing the adhesin in the low-affinity conformation and matching the loop conformation in the Abp2D crystal structure.

**Figure 5:**
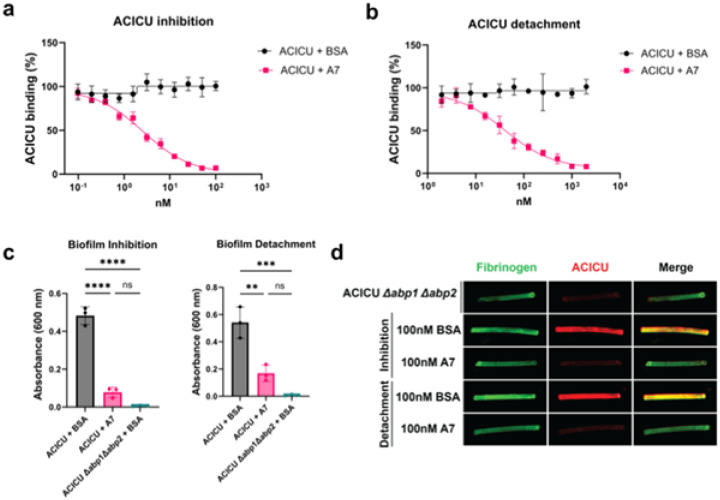
*In cellulo* characterization of Abp minibinder A7. **(A)** Inhibition ELISA results for minibinder A7 (IC50=2.6 nM; 95% CI: 1.8–3.0 nM) with *A. baumannii* ACICU. Each experiment includes three biological replicates. Error bars show standard deviation. **(B)** Detachment ELISA results for A7 (IC50=40.0 nM; 95% CI: 25.5–52.1 nM) and *A. baumannii* ACICU. Each experiment includes three replicates. Error bars show standard deviation. **(C)** A7 significantly inhibits the formation of biofilms (left) and disperses existing biofilms (right; n=3 independent replicates). One-way ANOVA test. ****P ≤ 0.0001, ***P ≤ 0.001, **P ≤ 0.01, *P ≤ 0.05. **(D)** Fluorescence imaging of fibrinogen-coated silicone tubing reveals differential adherence of *A. baumannii* ACICU after pretreatment with or dispersion by A7 or a BSA control. The catheters in this panel were captured in a single imaging session and uniform processing was applied to the entire image. Image representative of 4 independent replicates (except for 100 nM BSA detachment; n=3).

To assess the ability of A7 to inhibit bacterial adhesion, we performed a bacterial ELISA assay with *A. baumannii* ACICU. When preincubated with ACICU prior to being added to the fibrinogen-coated ELISA plate, A7 but not a BSA control significantly inhibited ACICU binding with an IC50 of 2.6 nM (95% CI: 1.8–3.0 nM; Fig. 5a). In a “detachment” ELISA, A7 successfully displaced adherent ACICU and exhibited an IC50 of 40.0 nM (95% CI: 25.5–52.1 nM; Fig. 5b). Since bacterial adhesins are known to contribute to biofilm formation (34), we also tested whether designed inhibitors could both prevent ACICU from forming biofilms and disperse preformed biofilms. Crystal violet staining of *A. baumannii* biofilms grown on PVC plates demonstrated that 1 μM of A7 could prevent the formation of ACICU biofilms, unlike a BSA control (p < 0.0001, one-way ANOVA; Fig. 5c). In addition, 1 μM of A7 but not a BSA control could almost completely disperse preformed ACICU biofilms after 2 hours of treatment (p=0.0021, one-way ANOVA), mimicking the phenotype of an ACICU Δabp1 Δabp2 double knockout strain (Fig. 5c; p=0.0003, one-way ANOVA).

To explore whether minibinder A7 could disrupt *A. baumannii* ACICU biofilms in a more *in vivo-*like context, inhibition and detachment studies were performed with fibrinogen-coated silicone catheters (34,37). After coating silicone tubing with fibrinogen, ACICU was allowed to bind in the presence of A7 or BSA. We also tested the ability of A7 and BSA to disperse ACICU that had already bound to the silicone catheter. Immunostaining the tubing revealed that despite fibrinogen coating each piece of tubing, 100 nM of A7 but not the BSA control significantly prevented ACICU attachment to and dispersed prebound bacteria from the tubing, mimicking the phenotype of the ACICU Δabp1 Δabp2 strain (Fig. 5d, fig. S13, p=0.0052, one-way ANOVA).

### Clinical models of adhesin inhibitors

**Figure 6:**
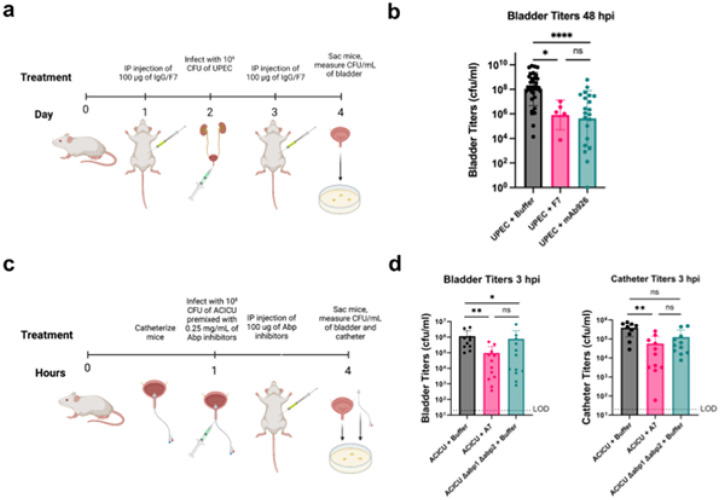
*In vivo* characterization of *de novo* designed adhesin inhibitors. **(A)** Schematic of UPEC UTI mouse model dosing regimen. Created with BioRender.com. **(B)** Titers of bacteria 48 hours post-infection (hpi) in the bladder after treatment with buffer (grey, n=34), minibinder F7 (pink, n=5), or monoclonal antibody (teal, n=21). Error bars show standard deviation. One-way ANOVA test. ****P ≤ 0.01, ***P ≤ 0.01, **P ≤ 0.01, *P ≤ 0.05. **(C)** Schematic of ACICU CAUTI mouse model dosing regimen. Mice were infected with 10^8^ cells of ACICU in a CAUTI model. Their catheters and bladders were processed for counting CFUs 3 hpi. Created with BioRender.com. **(D)** Bladder (left) and catheter (right) titers of ACICU and ACICU Δabp1 Δabp2 3 hpi. ACICU were treated with buffer control (gray, n=9) or A7 (pink, n=12). ACICU Δabp1 Δabp2 were treated with buffer (teal, n=11). Error bars show standard deviation. One-way ANOVA test. **P ≤ 0.01, *P ≤ 0.05.

We next explored whether these adhesin inhibitors would function *in vivo* in clinical models of UTI. To test anti-FimH minibinder F7’s ability to combat uncomplicated UTI, mice were injected intraperitoneally with either 100 μg of the minibinder, mAb926, or a buffer-only control 24 hours prior to bacterial challenge. Mice were then challenged with model uropathogenic *E. coli* strain CFT073 from the clonal group ST73 (10^6^ CFU), and a second dose of protein was administered by intraperitoneal (IP) injection 24 hours after infection. Forty-eight hours post-infection, bacterial burden in the bladder was quantified (Fig. 6a). F7-treated mice showed a significant (~2 log) reduction in bacterial bladder titers compared to the mock control group (p = 0.034, one-way ANOVA) and reached similar levels of reduction as the antibody-treated group (Fig. 6b).

We next tested anti-Abp minibinder A7 in a mouse model of CAUTI. Mice were catheterized and immediately infected with *A. baumannii* ACICU (10^8^ CFU) premixed with 0.25 mg/mL of minibinder or buffer. After 1 hour, mice were treated with IP injections of 100 μg of A7 or a buffer-only control. After 3 hours, bacterial burden in the bladder and catheter of each mouse was quantified (Fig. 6c). Mice that were treated with A7 exhibited significantly reduced bacterial loads in the bladder (p = 0.0035, one-way ANOVA) and catheter (p = 0.0014, one-way ANOVA) compared to mock-treated controls (Fig. 6d). This reduction in bacterial burden aligns with previous studies showing only modest (~1 log) attenuation of bacterial burden by ACICU Δabp1 Δabp2, suggesting alternative adhesion mechanisms may be involved in this mouse model (34). This proof of concept experiment lays the ground for future antibiotic-sparing strategies against pathogens of great concern.

### Optimization of adhesin inhibitors

We explored two routes to generating higher affinity designs. First, we used ProteinMPNN to resample sequences based on the designed backbone of minibinder F7, and selected variants with improved AF2 Initial Guess scores; this led to the identification of a new minibinder (C8) exhibiting ~10-fold higher affinity for FimH (LAS K_d_=15.0nM; HAS K_d_=243nM) and more than ~30-fold greater inhibitory activity in an inhibition ELISA with an IC50 of 37 nM (95% CI: 31–47 nM; Fig. S14, S15). Second, to enhance affinity via avidity effects, minibinder A7 was fused to de novo designed homo-oligomeric domains (38), generating multivalent binders. Oligomeric constructs such as oligomer C11 displayed significantly increased binding affinity to both adhesins, with K_d_s in the picomolar range for Abp1D and Abp2D (fig. S16). In cellulo, oligomer C11 exhibits a significantly lower inhibition ELISA IC50 (0.36 nM) than A7 (95% CI: 0.15–0.61 nM; fig. S17). Such affinity and avidity-increasing strategies should be generally applicable for further improving the *in vivo* efficacy of designed anti-adhesin minibinders.

## Discussion

In this study we show that *de novo* protein design tools can be employed to design potent and specific minibinder inhibitors of bacterial adhesins, virulence factors critical for colonization and biofilm formation in a broad range of bacterial infections, including UPEC UTI and *A. baumannii* CAUTI. The choice of target was key to *in vivo* success, as the extracellular nature of these adhesins made them readily accessible to the designed proteins. Being able to specifically target minibinders to the substrate binding epitope using RFdiffusion with hotspot conditioning led to neutralizing binders without the need for antibody screening or knowledge of the native substrates. The conversion of FimH HAS to LAS by minibinder F7 shows that computational design allows not only the selection of target epitope but also target conformation. Targeting the substrate binding pocket of these adhesins should make it unlikely that resistance to these binders will arise quickly, as any mutation to the binding pocket may also disrupt adhesion. The specificity of these designed proteins for their targets should enable selective depletion of these pathogens without affecting commensal microbiota and limit pressure among the broader bacterial community to evolve resistance to the minibinders (39). Future studies should explore whether resistance can be evolved to escape these minibinders and how they affect the broader microbiome.

Future development of the adhesion-neutralizing therapeutics demonstrated here will require characterization and possibly additional optimization of the pharmacokinetics and bioavailability of these designed proteins. Blocking adhesin activity using minibinders has potential utility against non-UTI bacterial pathogens such as *Staphylococcus aureus*, *Streptococcus pneumoniae*, and *Salmonella* (40,41). The strategies developed here should be applicable to combat the adhesion and colonization of pathogens beyond bacteria, including fungi and amoebae (42–44).

## Supplementary Material

Supplementary Files

This is a list of supplementary files associated with this preprint. Click to download.

- AdhesinpaperNatureMicroSubmissionSupplement.docx

## Data Availability

The crystal structures have been deposited to the Protein Data Bank (PDB) with accession codes 9Q1V (FimH-F7), 9Q1H (Abp2D-A7). All other data are available in the main text or the supplementary materials
